# The genome sequence of an ichneumonid wasp, *Cylloceria caligata* (Gravenhorst, 1829)

**DOI:** 10.12688/wellcomeopenres.22776.1

**Published:** 2024-08-14

**Authors:** Jaswinder Boparai, Gavin R. Broad, Natural History Museum Genome Acquisition Lab, Darwin Tree of Life Barcoding collective, Wellcome Sanger Institute Tree of Life Management, Samples and Laboratory team, Wellcome Sanger Institute Scientific Operations: Sequencing Operations, Wellcome Sanger Institute Tree of Life Core Informatics team, Tree of Life Core Informatics collective, Darwin Tree of Life Consortium

**Affiliations:** 1 Independent researcher, Brierley Hill, England, UK; 2 Natural History Museum, London, England, UK

**Keywords:** Cylloceria caligata, ichneumonid wasp, genome sequence, chromosomal, Hymenoptera

## Abstract

We present a genome assembly from an individual male *Cylloceria caligata* (an ichneumonid wasp; Arthropoda; Insecta; Hymenoptera; Ichneumonidae). The genome sequence spans 596.20 megabases. Most of the assembly is scaffolded into 9 chromosomal pseudomolecules. The mitochondrial genome has also been assembled and is 33.21 kilobases in length.

## Species taxonomy

Eukaryota; Opisthokonta; Metazoa; Eumetazoa; Bilateria; Protostomia; Ecdysozoa; Panarthropoda; Arthropoda; Mandibulata; Pancrustacea; Hexapoda; Insecta; Dicondylia; Pterygota; Neoptera; Endopterygota; Hymenoptera; Apocrita; Ichneumonoidea; Ichneumonidae; Cylloceriinae; *Cylloceria*; *Cylloceria calig*ata (Gravenhorst, 1829) (NCBI:txid2881813).

## Background

*Cylloceria caligata* is an ichneumonid wasp in the subfamily Cylloceriinae. There has been little consensus over the composition of the subfamily, however, four genera have been assigned to Cylloceriinae and three of these occur in Britain. Seven species, representing *Allomacrus*, *Cylloceria* and *Hyperacmus*, are known from Britain ( Broad *et al.*, 2018), although not all of the species have been certainly identified (GRB, pers. obs.). Unlike other British *Cylloceria*, *C. caligata* have red coxae and the female has a relatively short ovipositor.

Little is known about the biology of the Cylloceriinae, but all species for which hosts are known are thought to be endophagous in Diptera ( Wahl, 1990). Species of *Cylloceria* have been reared from the larvae of *Tipula* (Diptera: Tipulidae) ( Broad *et al*., 2018) and *Cylloceria aino* Uchida, 1928, from the Russian Far East, was reared from a cocoon of an unidentified Tipulidae ( Humala, 2002). The rather long ovipositor is used to contact and oviposit in cranefly larvae in concealment.

Cylloceriinae is one of a monophyletic group of (currently) nine subfamilies ( Gauld, 1991; Quicke *et al.*, 2009), given the informal name ‘Pimpliformes’ when first proposed by Wahl and Gauld (1998). Within the Pimpliformes, the recent molecular phylogenetic study by Spasojevic *et al*. (2021) recognised the close association of Cylloceriinae with Diacritinae and Orthocentrinae, the close association supported by their biology, where known, as koinobiont endoparasitoids of Diptera. However, based on both molecular and morphological evidence, the boundaries of these subfamilies are difficult, and Spasojevic *et al*. (2021) suggested they might be synonymised in the future.

Adults of *C. caligata* have a forewing length of ∼6-8mm and are on the wing from mid-summer through to late autumn. Locally common, they can be found flying over low vegetation in damp habitats, e.g., along the banks of streams or damp areas of woodlands. There are, however, no reliable host records. *Cylloceria caligata* is trans-Palaearctic in distribution (summarised by Yu *et al.*, 2016) and is one of three species of *Cylloceria* currently recorded from Britain, easily distinguished from the two others by the red hind coxae ( Humala, 2002). *Cylloceria* males have characteristic semi-circular emarginations on flagellar segments 3 and 4. Keys to European species of Cylloceriinae, including *Cylloceria*, can be found in Humala ( 2002, 2003, 2007).

To investigate the internal phylogenetic relationships of Ichneumonidae, several genes of some *Cylloceria* species have been sequenced previously ( Bennett *et al.*, 2019; Klopfstein *et al.*, 2019; Quicke *et al.*, 2009). Klopfstein *et al*. (2019) focused on the phylogenetic relationships of Pimpliformes and included *Cylloceria* cf. *caligata* in their analyses. However, this is the first chromosome-level genome sequence of a species of the subfamily Cylloceriinae.

## Genome sequence report

**Figure 1.**
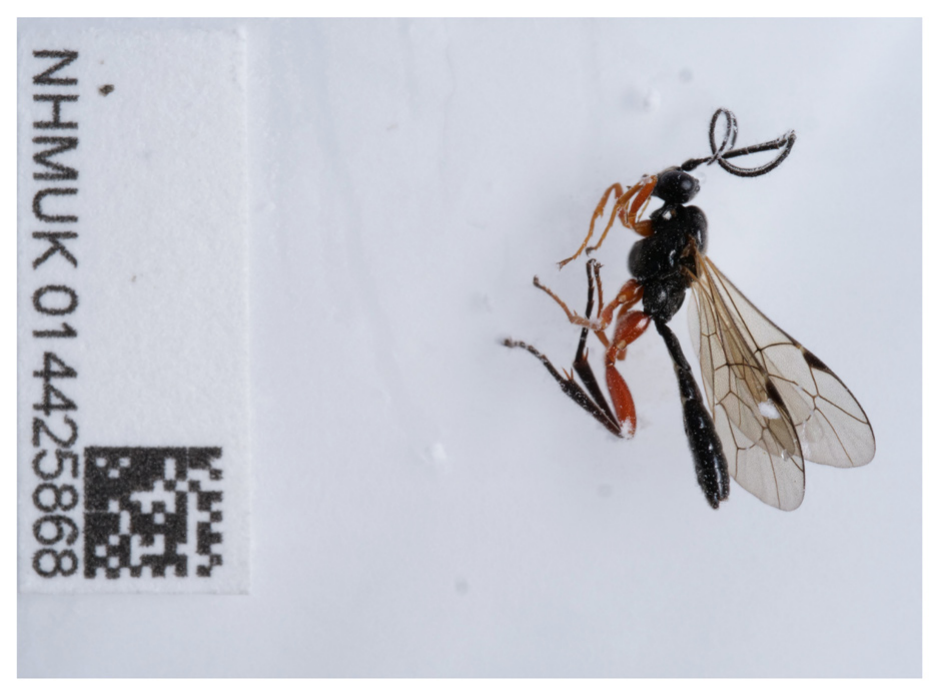
Photograph of the *Cylloceria caligata* (iyCylCali1) specimen used for genome sequencing.

The genome of an adult male *Cylloceria caligata* ( Figure 1) was sequenced using Pacific Biosciences single-molecule HiFi long reads, generating a total of 21.44 Gb (gigabases) from 2.25 million reads, providing approximately 37-fold coverage. Primary assembly contigs were scaffolded with chromosome conformation Hi-C data, which produced 117.14 Gbp from 775.74 million reads, yielding an approximate coverage of 196-fold. Specimen and sequencing information is summarised in Table 1.

**Table 1.** Specimen and sequencing data for *Cylloceria caligata*.

Project information			
**Study title**	Cylloceria caligata		
**Umbrella BioProject**	PRJEB66049		
**Species**	*Cylloceria caligata*		
**BioSample**	SAMEA14448408		
**NCBI taxonomy ID**	2881813		
Specimen information			
**Technology**	**ToLID**	**BioSample accession**	**Organism part**
**PacBio long read** **sequencing**	iyCylCali1	SAMEA14448783	head and thorax
**Hi-C sequencing**	iyCylCali1	SAMEA14448783	head and thorax
Sequencing information			
**Platform**	**Run accession**	**Read count**	**Base count (Gb)**
**Hi-C Illumina NovaSeq** **6000**	ERR12071262	7.76e+08	117.14
**PacBio Sequel IIe**	ERR12055581	2.25e+06	21.44

**Figure 2.**
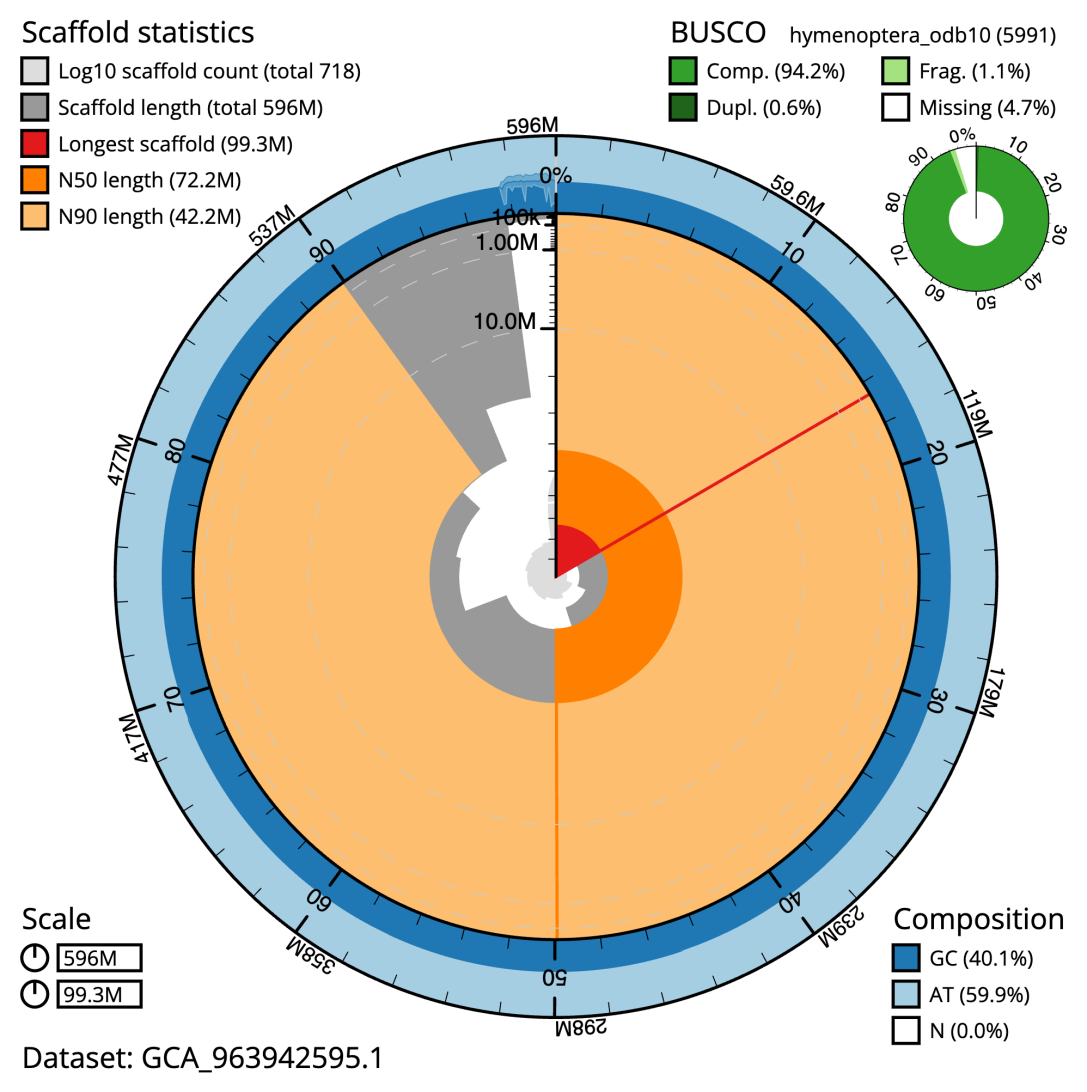
Genome assembly of *Cylloceria caligata*, iyCylCali1.1: metrics. The BlobToolKit snail plot shows N50 metrics and BUSCO gene completeness. The main plot is divided into 1,000 size-ordered bins around the circumference with each bin representing 0.1% of the 596,283,113 bp assembly. The distribution of scaffold lengths is shown in dark grey with the plot radius scaled to the longest scaffold present in the assembly (99,311,508 bp, shown in red). Orange and pale-orange arcs show the N50 and N90 scaffold lengths (72,214,643 and 42,159,388 bp), respectively. The pale grey spiral shows the cumulative scaffold count on a log scale with white scale lines showing successive orders of magnitude. The blue and pale-blue area around the outside of the plot shows the distribution of GC, AT and N percentages in the same bins as the inner plot. A summary of complete, fragmented, duplicated and missing BUSCO genes in the hymenoptera_odb10 set is shown in the top right. An interactive version of this figure is available at https://blobtoolkit.genomehubs.org/view/Cylloceria_caligata/dataset/GCA_963942595.1/snail.

**Figure 3.**
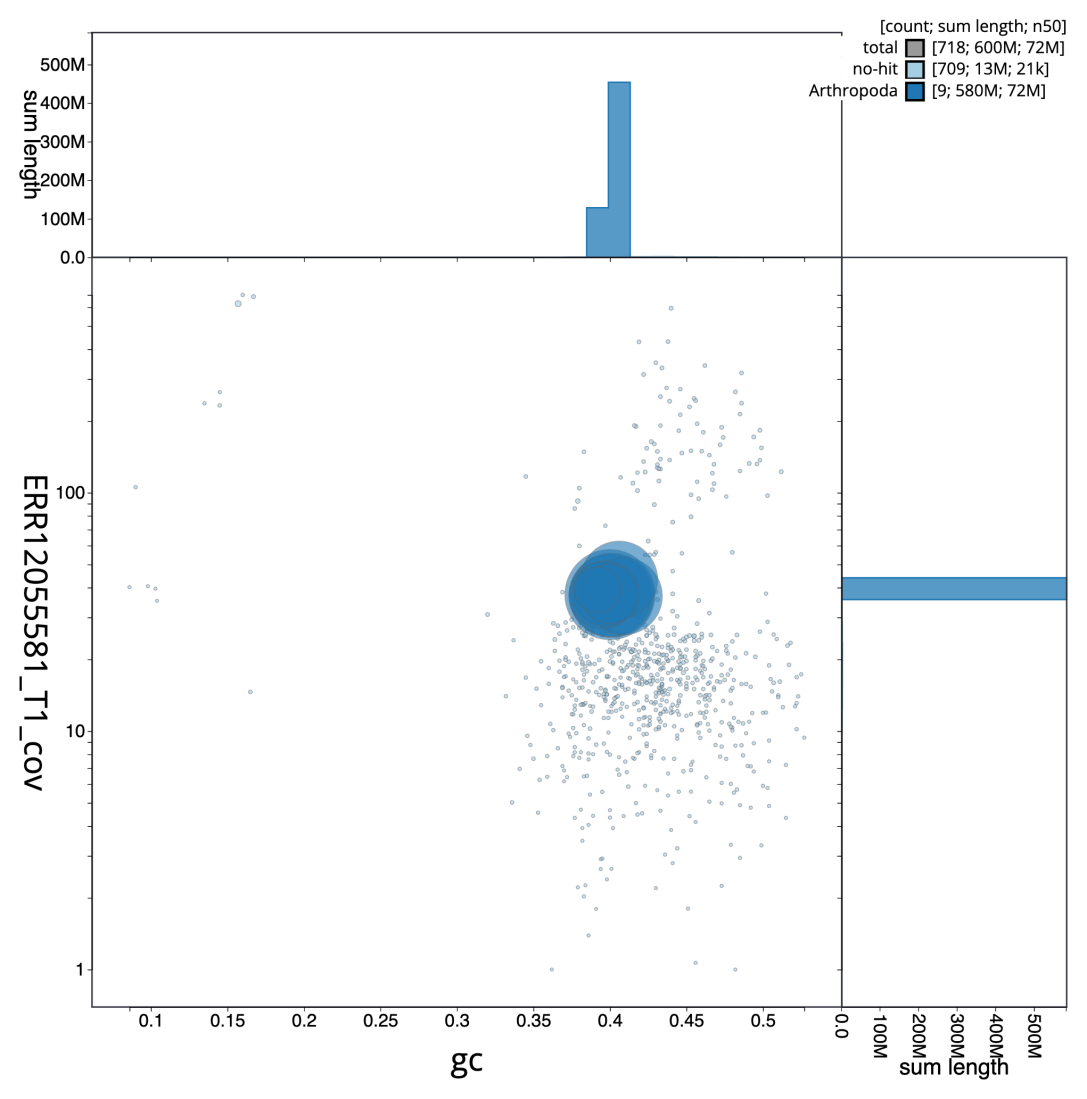
Genome assembly of *Cylloceria caligata*, iyCylCali1.1: BlobToolKit GC-coverage plot. Sequences are coloured by phylum. Circles are sized in proportion to sequence length. Histograms show the distribution of sequence length sum along each axis. An interactive version of this figure is available at https://blobtoolkit.genomehubs.org/view/Cylloceria_caligata/dataset/GCA_963942595.1/blob.

**Figure 4.**
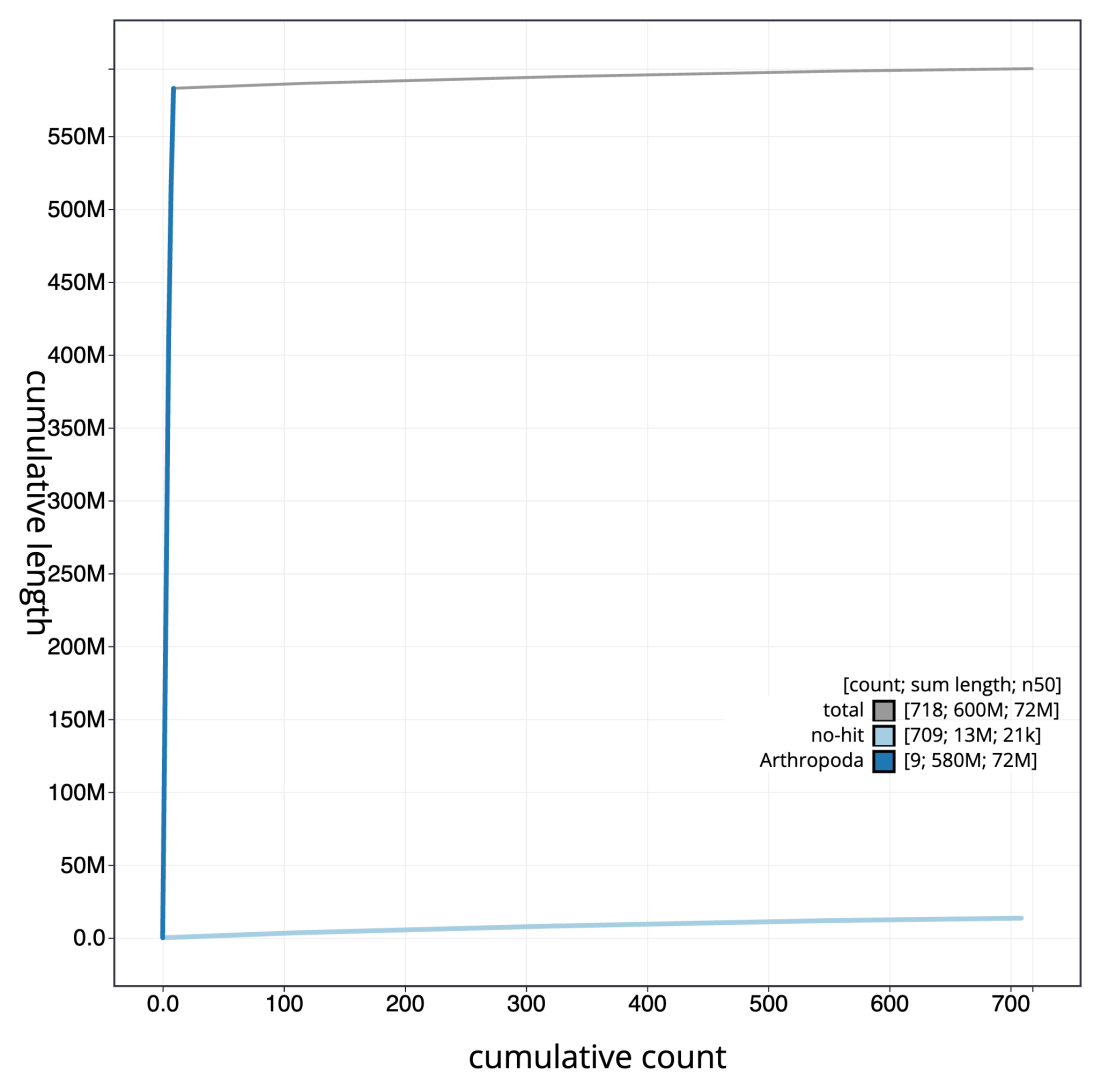
Genome assembly of *Cylloceria caligata* iyCylCali1.1: BlobToolKit cumulative sequence plot. The grey line shows cumulative length for all sequences. Coloured lines show cumulative lengths of sequences assigned to each phylum using the buscogenes taxrule. An interactive version of this figure is available at https://blobtoolkit.genomehubs.org/view/Cylloceria_caligata/dataset/GCA_963942595.1/cumulative.

**Figure 5.**
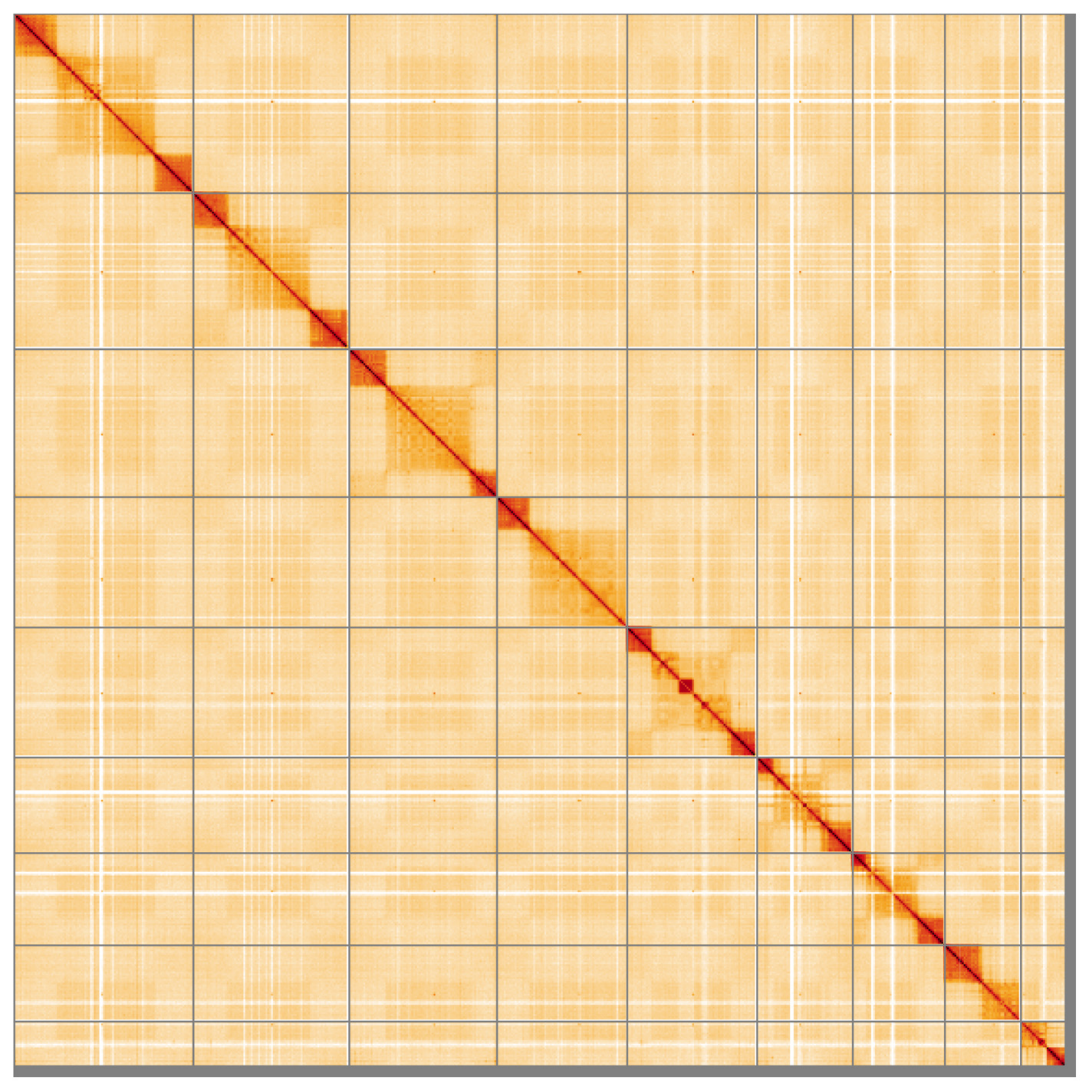
Genome assembly of *Cylloceria caligata* iyCylCali1.1: Hi-C contact map of the iyCylCali1.1 assembly, visualised using HiGlass. Chromosomes are shown in order of size from left to right and top to bottom. An interactive version of this figure may be viewed at https://genome-note-higlass.tol.sanger.ac.uk/l/?d=M_eaqlzFTsG-sxCxFQda2w.

Manual assembly curation corrected 298 missing joins or mis-joins, reducing the scaffold number by 19.05%, and increasing the scaffold N50 by 2.32%. The final assembly has a total length of 596.20 Mb in 717 sequence scaffolds with a scaffold N50 of 72.2 Mb ( Table 2), and 1,291 gaps. The snail plot in Figure 2 provides a summary of the assembly statistics, while the distribution of assembly scaffolds on GC proportion and coverage is shown in Figure 3. The cumulative assembly plot in Figure 4 shows curves for subsets of scaffolds assigned to different phyla. Most (97.74%) of the assembly sequence was assigned to 9 chromosomal-level scaffolds. Chromosome-scale scaffolds confirmed by the Hi-C data are named in order of size ( Figure 5; Table 3). the exact order and orientation of the repeat region on chromosome 5 (29.4 Mbp– 37.4 Mbp) is unknown. The mitochondrial genome was also assembled and can be found as a contig within the multifasta file of the genome submission.

The estimated Quality Value (QV) of the final assembly is 59.5 with *k*-mer completeness of 99.99%, and the assembly has a BUSCO v5.4.3 completeness of 94.0% (single = 93.4%, duplicated = 0.6%), using the hymenoptera_odb10 reference set ( *n* = 5,991).

**Table 2.** Genome assembly data for *Cylloceria caligata*, iyCylCali1.1.

Genome assembly		
Assembly name	iyCylCali1.1	
Assembly accession	GCA_963942595.1	
Span (Mb)	596.20	
Number of contigs	2,009	
Contig N50 length (Mb)	0.9	
Number of scaffolds	717	
Scaffold N50 length (Mb)	72.2	
Longest scaffold (Mb)	99.31	
Assembly metrics *		*Benchmark*
Consensus quality (QV)	59.5	*≥ 50*
*k*-mer completeness	99.99%	*≥ 95%*
BUSCO **	C:94.0%[S:93.4%,D:0.6%], F:1.6%,M:4.4%,n:5,991	*C ≥ 95%*
Percentage of assembly mapped to chromosomes	97.74%	*≥ 95%*
Sex chromosomes	None	*localised homologous pairs*
Organelles	Mitochondrial genome: 33.21 kb	*complete single alleles*

* Assembly metric benchmarks are adapted from column VGP-2020 of “Table 1: Proposed standards and metrics for defining genome assembly quality” from Rhie *et al.* (2021). ** BUSCO scores based on the hymenoptera_odb10 BUSCO set using version 5.4.3. C = complete [S = single copy, D = duplicated], F = fragmented, M = missing, n = number of orthologues in comparison. A full set of BUSCO scores is available at https://blobtoolkit.genomehubs.org/view/Cylloceria_caligata/dataset/GCA_963942595.1/busco.

**Table 3.** Chromosomal pseudomolecules in the genome assembly of *Cylloceria caligata*, iyCylCali1.

INSDC accession	Name	Length (Mb)	GC%
OZ012703.1	1	99.31	40.0
OZ012704.1	2	86.42	40.0
OZ012705.1	3	81.92	40.0
OZ012706.1	4	72.21	41.0
OZ012707.1	5	71.99	40.5
OZ012708.1	6	53.01	39.5
OZ012709.1	7	51.08	39.5
OZ012710.1	8	42.16	40.0
OZ012711.1	9	24.72	39.0
OZ012712.1	MT	0.03	17.0

Metadata for specimens, BOLD barcode results, spectra estimates, sequencing runs, contaminants and pre-curation assembly statistics are given at https://links.tol.sanger.ac.uk/species/2881813.

## Methods

### Sample acquisition

A male *Cylloceria caligata* (specimen ID NHMUK014425868, ToLID iyCylCali1) was collected from Bell Coppice, Shropshire, UK (latitude 52.37, longitude –2.41) using a sweep net. The specimen was collected and identified by Jaswinder Boparai (independent researcher) and sent to Gavin Broad (Natural History Museum). The specimen was preserved by dry-freezing at –80°C.

The initial identification was verified by an additional DNA barcoding process according to the framework developed by Twyford *et al*. (2024). A small sample was dissected from the specimen and stored in ethanol, while the remaining parts of the specimen were shipped on dry ice to the Wellcome Sanger Institute (WSI). The tissue was lysed, the COI marker region was amplified by PCR, and amplicons were sequenced and compared to the BOLD database, confirming the species identification ( Crowley *et al.*, 2023). Following whole genome sequence generation, the relevant DNA barcode region was also used alongside the initial barcoding data for sample tracking at the WSI ( Twyford *et al.*, 2024). The standard operating procedures for Darwin Tree of Life barcoding have been deposited on protocols.io ( Beasley *et al.*, 2023).

### Nucleic acid extraction

The workflow for high molecular weight (HMW) DNA extraction at the WSI Tree of Life Core Laboratory includes a sequence of core procedures: sample preparation; sample homogenisation, DNA extraction, fragmentation, and clean-up. In sample preparation, the iyCylCali1 sample was weighed and dissected on dry ice ( Jay *et al.*, 2023). Tissue from the head and thorax was homogenised using a PowerMasher II tissue disruptor ( Denton *et al.*, 2023a).

HMW DNA was extracted at the WSI Scientific Operations core using the Automated MagAttract v2 protocol ( Oatley *et al.*, 2023). The DNA was sheared into an average fragment size of 12–20 kb in a Megaruptor 3 system ( Bates *et al.*, 2023). Sheared DNA was purified by solid-phase reversible immobilisation ( Strickland *et al.*, 2023): in brief, the method employs AMPure PB beads to eliminate shorter fragments and concentrate the DNA. The concentration of the sheared and purified DNA was assessed using a Nanodrop spectrophotometer and Qubit Fluorometer using the Qubit dsDNA High Sensitivity Assay kit. Fragment size distribution was evaluated by running the sample on the FemtoPulse system.

Protocols developed by the WSI Tree of Life laboratory are publicly available on protocols.io ( Denton *et al.*, 2023b).

### Sequencing

Pacific Biosciences HiFi circular consensus DNA sequencing libraries were constructed according to the manufacturers’ instructions. DNA sequencing was performed by the Scientific Operations core at the WSI on a Pacific Biosciences Sequel IIe instrument. Hi-C data were also generated from head and thorax tissue of iyCylCali1 using the Arima-HiC v2 kit. The Hi-C sequencing was performed using paired-end sequencing with a read length of 150 bp on the Illumina NovaSeq 6000 instrument.

### Genome assembly, curation and evaluation

***Assembly***

The original assembly of HiFi reads was performed using Hifiasm ( Cheng *et al.*, 2021) with the --primary option. Hi-C reads are further mapped with bwamem2 ( Vasimuddin *et al.*, 2019) to the primary contigs, which are further scaffolded using the provided Hi-C data ( Rao *et al.*, 2014) in YaHS ( Zhou *et al.*, 2023) using the --break option. Scaffolded assemblies are evaluated using Gfastats ( Formenti *et al.*, 2022), BUSCO ( Manni *et al.*, 2021) and MERQURY.FK ( Rhie *et al.*, 2020).

The mitochondrial genome was assembled using MitoHiFi ( Uliano-Silva *et al.*, 2023), which runs MitoFinder ( Allio *et al.*, 2020) and uses these annotations to select the final mitochondrial contig and to ensure the general quality of the sequence.

***Assembly curation***

The assembly was decontaminated using the Assembly Screen for Cobionts and Contaminants (ASCC) pipeline (article in preparation). Flat files and maps used in curation were generated in TreeVal ( Pointon *et al.*, 2023). Manual curation was primarily conducted using PretextView ( Harry, 2022), with additional insights provided by JBrowse2 ( Diesh *et al.*, 2023) and HiGlass ( Kerpedjiev *et al.*, 2018). Scaffolds were visually inspected and corrected as described by Howe *et al*. (2021). Any identified contamination, missed joins, and mis-joins were corrected, and duplicate sequences were tagged and removed. The entire process is documented at https://gitlab.com/wtsi-grit/rapid-curation (article in preparation).

***Evaluation of the final assembly***

The final assembly was post-processed and evaluated with the three Nextflow ( Di Tommaso *et al.*, 2017) DSL2 pipelines “sanger-tol/readmapping” ( Surana *et al.*, 2023a), “sanger-tol/genomenote” ( Surana *et al.*, 2023b), and “sanger-tol/blobtoolkit” ( Muffato *et al.*, 2024). The pipeline sanger-tol/readmapping aligns the Hi-C reads with bwa-mem2 ( Vasimuddin *et al.*, 2019) and combines the alignment files with SAMtools ( Danecek *et al.*, 2021). The sanger-tol/genomenote pipeline transforms the Hi-C alignments into a contact map with BEDTools ( Quinlan & Hall, 2010) and the Cooler tool suite ( Abdennur & Mirny, 2020), which is then visualised with HiGlass ( Kerpedjiev *et al.*, 2018). It also provides statistics about the assembly with the NCBI datasets ( Sayers *et al.*, 2024) report, computes *k*-mer completeness and QV consensus quality values with FastK and MERQURY.FK, and a completeness assessment with BUSCO ( Manni *et al.*, 2021).

The sanger-tol/blobtoolkit pipeline is a Nextflow port of the previous Snakemake Blobtoolkit pipeline ( Challis *et al.*, 2020). It aligns the PacBio reads with SAMtools and minimap2 ( Li, 2018) and generates coverage tracks for regions of fixed size. In parallel, it queries the GoaT database ( Challis *et al.*, 2023) to identify all matching BUSCO lineages to run BUSCO ( Manni *et al.*, 2021). For the three domain-level BUSCO lineage, the pipeline aligns the BUSCO genes to the Uniprot Reference Proteomes database ( Bateman *et al.*, 2023) with DIAMOND ( Buchfink *et al.*, 2021) blastp. The genome is also split into chunks according to the density of the BUSCO genes from the closest taxonomically lineage, and each chunk is aligned to the Uniprot Reference Proteomes database with DIAMOND blastx. Genome sequences that have no hit are then chunked with seqtk and aligned to the NT database with blastn ( Altschul *et al.*, 1990). All those outputs are combined with the blobtools suite into a blobdir for visualisation.

The genome assembly and evaluation pipelines were developed using the nf-core tooling ( Ewels *et al.*, 2020), use MultiQC ( Ewels *et al.*, 2016), and make extensive use of the Conda package manager, the Bioconda initiative ( Grüning *et al.*, 2018), the Biocontainers infrastructure ( da Veiga Leprevost *et al.*, 2017), and the Docker ( Merkel, 2014) and Singularity ( Kurtzer *et al.*, 2017) containerisation solutions.

Table 4 contains a list of relevant software tool versions and sources.

**Table 4.** Software tools: versions and sources.

Software tool	Version	Source
BEDTools	2.30.0	https://github.com/arq5x/bedtools2
BLAST	2.14.0	ftp://ftp.ncbi.nlm.nih.gov/blast/executables/blast+/
BlobToolKit	4.3.7	https://github.com/blobtoolkit/blobtoolkit
BUSCO	5.4.3 and 5.5.0	https://gitlab.com/ezlab/busco
bwa-mem2	2.2.1	https://github.com/bwa-mem2/bwa-mem2
Cooler	0.8.11	https://github.com/open2c/cooler
DIAMOND	2.1.8	https://github.com/bbuchfink/diamond
fasta_windows	0.2.4	https://github.com/tolkit/fasta_windows
FastK	427104ea91c78c3b8b8b49f1a7d6bbeaa869ba1c	https://github.com/thegenemyers/FASTK
Gfastats	1.3.6	https://github.com/vgl-hub/gfastats
GoaT CLI	0.2.5	https://github.com/genomehubs/goat-cli
Hifiasm	0.16.1	https://github.com/chhylp123/hifiasm
HiGlass	44086069ee7d4d3f6f3f0012569789ec138f42b84a a44357826c0b6753eb28de	https://github.com/higlass/higlass
Merqury.FK	d00d98157618f4e8d1a9190026b19b471055b22e	https://github.com/thegenemyers/MERQURY.FK
MitoHiFi	2	https://github.com/marcelauliano/MitoHiFi
MultiQC	1.14, 1.17, and 1.18	https://github.com/MultiQC/MultiQC
NCBI Datasets	15.12.0	https://github.com/ncbi/datasets
Nextflow	23.04.0-5857	https://github.com/nextflow-io/nextflow
PretextView	0.2	https://github.com/sanger-tol/PretextView
samtools	1.16.1, 1.17, and 1.18	https://github.com/samtools/samtools
sanger-tol/ascc	-	https://github.com/sanger-tol/ascc
sanger-tol/genomenote	1.1.1	https://github.com/sanger-tol/genomenote
sanger-tol/readmapping	1.2.1	https://github.com/sanger-tol/readmapping
Seqtk	1.3	https://github.com/lh3/seqtk
Singularity	3.9.0	https://github.com/sylabs/singularity
TreeVal	1.0.0	https://github.com/sanger-tol/treeval
YaHS	1.1a.2	https://github.com/c-zhou/yahs

### Wellcome Sanger Institute – Legal and Governance

The materials that have contributed to this genome note have been supplied by a Darwin Tree of Life Partner. The submission of materials by a Darwin Tree of Life Partner is subject to the **‘Darwin Tree of Life Project Sampling Code of Practice’**, which can be found in full on the Darwin Tree of Life website here. By agreeing with and signing up to the Sampling Code of Practice, the Darwin Tree of Life Partner agrees they will meet the legal and ethical requirements and standards set out within this document in respect of all samples acquired for, and supplied to, the Darwin Tree of Life Project. 

Further, the Wellcome Sanger Institute employs a process whereby due diligence is carried out proportionate to the nature of the materials themselves, and the circumstances under which they have been/are to be collected and provided for use. The purpose of this is to address and mitigate any potential legal and/or ethical implications of receipt and use of the materials as part of the research project, and to ensure that in doing so we align with best practice wherever possible. The overarching areas of consideration are:

•   Ethical review of provenance and sourcing of the material

•   Legality of collection, transfer and use (national and international)

Each transfer of samples is further undertaken according to a Research Collaboration Agreement or Material Transfer Agreement entered into by the Darwin Tree of Life Partner, Genome Research Limited (operating as the Wellcome Sanger Institute), and in some circumstances other Darwin Tree of Life collaborators.

## Data Availability

European Nucleotide Archive: *Cylloceria caligata*. Accession number PRJEB66049; https://identifiers.org/ena.embl/PRJEB66049 ( Wellcome Sanger Institute, 2024). The genome sequence is released openly for reuse. The *Cylloceria caligata* genome sequencing initiative is part of the Darwin Tree of Life (DToL) project. All raw sequence data and the assembly have been deposited in INSDC databases. The genome will be annotated using available RNA-Seq data and presented through the Ensembl pipeline at the European Bioinformatics Institute. Raw data and assembly accession identifiers are reported in Table 1 and Table 2.

## Author information

Members of the Natural History Museum Genome Acquisition Lab are listed here: https://doi.org/10.5281/zenodo.12159242.

Members of the Darwin Tree of Life Barcoding collective are listed here: https://doi.org/10.5281/zenodo.12158331

Members of the Wellcome Sanger Institute Tree of Life Management, Samples and Laboratory team are listed here: https://doi.org/10.5281/zenodo.12162482.

Members of Wellcome Sanger Institute Scientific Operations: Sequencing Operations are listed here: https://doi.org/10.5281/zenodo.12165051.

Members of the Wellcome Sanger Institute Tree of Life Core Informatics team are listed here: https://doi.org/10.5281/zenodo.12160324.

Members of the Tree of Life Core Informatics collective are listed here: https://doi.org/10.5281/zenodo.12205391.

Members of the Darwin Tree of Life Consortium are listed here: https://doi.org/10.5281/zenodo.4783558.
