# Supplementary material for: The genome sequence of an ichneumonid wasp, Cylloceria caligata (Gravenhorst, 1829)
Source: Wellcome Open Res. 2024 Aug 14;9:478. [Version 1] doi: 10.12688/wellcomeopenres.22776.1 (PMC11489849; doi:10.12688/wellcomeopenres.22776.1)
Supplement: Peer review history

# Reviewer report: Reviewer response for version 1

**DOI:** 10.21956/wellcomeopenres.25080.r98902

**Published:** 2024-10-18

**Authors:** Rosanna Giordano

**Affiliations:** 1 Florida International University, Miami, Florida, USA

The manuscript presents the genome of *Cylloceria caligata*, as such it significantly advances our knowledge about a group of organisms about which little is known.

 The manuscript has a good background review of the current taxonomic status of the subfamily Cylloceriinae and the uncertainty associated with its relationship to other subfamilies within the group. All of which have been described as koinobiont endoparasitoids of Diptera. It also briefly describes the habitat it can be found in the UK. While individual genes have been sequenced for some Cylloceria species aiming to discern their phylogenetic relationships, this is the first genome for a member of the Cylloceriinae subfamily. 

 The methods are outlined clearly and in sufficient detail to allow the reproduction of this work.  The tools and methods used to generate the data have well specified.  The quality of the genome is high with a BUSCO completeness score of 94% and k-mer completness of 99.99%. The combination of PacBio and Hi-C reads has facilitated the assembly of a good quality genome.

Are sufficient details of methods and materials provided to allow replication by others?

Yes

Is the rationale for creating the dataset(s) clearly described?

Yes

Are the datasets clearly presented in a useable and accessible format?

Yes

Are the protocols appropriate and is the work technically sound?

Yes

Reviewer Expertise:

Entomology, Molecular Biology, Symbionts

I confirm that I have read this submission and believe that I have an appropriate level of expertise to confirm that it is of an acceptable scientific standard.

# Reviewer report: Reviewer response for version 1

**DOI:** 10.21956/wellcomeopenres.25080.r98911

**Published:** 2024-09-16

**Authors:** Eric Ganko

**Affiliations:** 1 Syngenta, Research Triangle Park, NC, USA

This paper details a genome sequence for *Cylloceria caligata*, a parasitoid wasp, via a combination of PacBio and Hi-C data. The rationale and pipelines for genome assembly have been thoroughly documented by the Darwin Tree of Life Consortium. The assembly quality is acceptable with good pseudomolule representation and reasonable BUSCO scores (especially at the higher taxa levels, i.e. insecta). 

**One minor note:** "Most of the assembly is scaffolded into 9 chromosomal pseudomolecules." -- you have the data to be precise here, per Table 2, 97.7% is connected to a chromosome.

Are sufficient details of methods and materials provided to allow replication by others?

Yes

Is the rationale for creating the dataset(s) clearly described?

Yes

Are the datasets clearly presented in a useable and accessible format?

Yes

Are the protocols appropriate and is the work technically sound?

Yes

Reviewer Expertise:

genomics, bioinformatics

I confirm that I have read this submission and believe that I have an appropriate level of expertise to confirm that it is of an acceptable scientific standard.
